# Evolution of Thermophilic Microbial Communities from a Deep-Sea Hydrothermal Chimney under Electrolithoautotrophic Conditions with Nitrate

**DOI:** 10.3390/microorganisms9122475

**Published:** 2021-11-30

**Authors:** Guillaume Pillot, Oulfat Amin Ali, Sylvain Davidson, Laetitia Shintu, Yannick Combet-Blanc, Anne Godfroy, Patricia Bonin, Pierre-Pol Liebgott

**Affiliations:** 1 Aix Marseille Université, Université de Toulon, CNRS, IRD, MIO UM 110, 13288 Marseille, France; guillaume.pillot@uni-bremen.de (G.P.); oulfat.aminali@gmail.com (O.A.A.); sylvain.davidson@mio.osupytheas.fr (S.D.); yannick.combet-blanc@mio.osupytheas.fr (Y.C.-B.); patricia.bonin@mio.osupytheas.fr (P.B.); 2 Aix Marseille Université, CNRS Centrale Marseille, iSm2, 13284 Marseille, France; laetitia.shintu@univ-amu.fr; 3 Laboratoire de Microbiologie des Environnements Extrêmes, Université de Bretagne Occidentale, CNRS, IFREMER, 29280 Plouzané, France; anne.godfroy@ifremer.fr

**Keywords:** microbial electrochemical systems, electroautotrophy, thermophily, deep-sea hydrothermal vent, nitrate

## Abstract

Recent studies have shown the presence of an abiotic electrical current across the walls of deep-sea hydrothermal chimneys, allowing the growth of electroautotrophic microbial communities. To understand the role of the different phylogenetic groups and metabolisms involved, this study focused on electrotrophic enrichment with nitrate as electron acceptor. The biofilm density, community composition, production of organic compounds, and electrical consumption were monitored by FISH confocal microscopy, qPCR, metabarcoding, NMR, and potentiostat measurements. A statistical analysis by PCA showed the correlation between the different parameters (qPCR, organic compounds, and electron acceptors) in three distinct temporal phases. In our conditions, the *Archaeoglobales* have been shown to play a key role in the development of the community as the first colonizers on the cathode and the first producers of organic compounds, which are then used as an organic source by heterotrophs. Finally, through subcultures of the community, we showed the development of a greater biodiversity over time. This observed phenomenon could explain the biodiversity development in hydrothermal contexts, where energy sources are transient and unstable.

## 1. Introduction

Deep-sea hydrothermal vents, discovered for the first time in 1977, harbor complex ecosystems sheltering extremophilic life forms [1,2]. These hydrothermal chimneys result from the infiltration of seawater into the seabed, which is then heated by an underlying magma chamber and reacts with surrounding minerals to produce a hot hydrothermal fluid (ca. 300–400 °C) rich in minerals and reduced compounds (H_2_, H_2_S, CH_4_). This hot, reduced, pressurized fluid moves back to the sea floor to precipitate in contact with the cold (ca. 2 °C) and oxidized surrounding seawater. These reactions form an area of intense mixing and severe thermal and chemical gradients, allowing for the development of a complex and extremophilic biosphere. Because of the high, sterilizing temperature of the hydrothermal fluid [3], the question of the identity of the first colonizers of these newly formed hydrothermal chimneys arises [3,4,5]. It is now accepted that, in the absence of organic matter and light, only chemolithoautotrophs coming from seawater or Earth’s crust can grow in the first stage of colonization. The colonizers would use the oxidation of reduced compounds from the hydrothermal fluid as an energy source and CO_2_ as a carbon source to produce organic matter. Some studies have attempted to study the microbial diversity in a newly formed chimney through the formation of a new chimney in mineral chambers and sampling units or on thermocouples placed in the hydrothermal fluid [4,5]. They highlighted the presence of *Desulfurococcaceae,* mainly members of the genus *Ignicoccus,* and its symbiont *Nanaoarchaeum* in addition to *Thermococcus* sp. and *Methanocaldococcus* spp. This primary production of organic matter would subsequently support the growth of a broad range of heterotrophs through the complex trophic chain and microbial interactions [6,7]. Besides enriching hyperthermophilic *Archaea*, recent studies have postulated that colonization of a newly erupted black smoker occurs by hyperthermophiles [8]. Two hyperthermophilic species—*Thermococcales* and *Methanococcales*—have been shown to swim to mineral surfaces, scan those surfaces to find optimal temperature conditions, and finally adhere directly to them through mechanisms that could be similar to those of the bacterial pili IV [9].

Most of the methods used to enrich these first colonizers in the laboratory have postulated the use of reduced compounds from the hydrothermal fluid as substrates. However, recent studies have shown the presence of an abiotic electrical current inside the chimney wall produced by chemical oxidation of hydrogen sulfide from hydrothermal fluid coupled with oxygen reduction from seawater. It has been hypothesized that this electrical current could be used as a direct energy source by electroactive microbes [10]. These microbes, extensively studied for 20 years, have the ability to perform extracellular electron transfer toward or from conductive support for their metabolism [11]. Microorganisms able to use direct electron flow as an energy source are called electrotrophs. They can take up electrons directly from the cathode, the electron donor, in a microbial electrochemical system (MES) for their energy and transfer it into a terminal electron acceptor. In the literature, many studies have aimed to enrich these electrotrophs within microbial communities sampled from various ecosystems [12,13,14,15,16] or to axenically grow them [17,18].

Recently, we have successfully enriched hyperthermophilic electrotrophs from hydrothermal vent samples [19]. Only the presence of CO_2_ as carbon source, an electron acceptor, and an abiotic electron flow fed by the MES cathode as energy source were sufficient to grow electroautotrophic microorganisms from hydrothermal chimneys. The community obtained in the electrotrophic biofilm was mainly composed of archaea from *Archaeoglobales* and *Thermococcales*, with the specific enrichment of other phylogenetic groups depending on the electron acceptor used. Most of the enriched microorganisms were affiliated within uncultured orders, families, genera, or species. This finding has raised some questions such as: is this first primary production enough to drive the development of more complex biodiversity through the food web and microbial interactions? In this latter context, nitrate was used as the electron acceptor due to its hypothesized central role in the hydrothermal origin of life theory [20], and its concentration up to 20 µM in hydrothermal vents [21].

The objective of this work was to study the evolution of the composition of the hyperthermophilic microbial community and organic products over time in order to identify the first colonizers and the metabolisms involved. This microbial community was cultured in electrolithoautotrophic conditions with a polarized electrode (cathode) as an electron donor and nitrate as the terminal electron acceptor. Thus, current density related to electrotrophic growth and nitrate consumption were monitored, the produced organic and gaseous compounds were measured, and dominant phylogenetic orders were quantified via qPCR. Afterward, we studied if this electrotrophic community could lead to a more complex ecosystem through two subsequent subcultures in an MES.

## 2. Materials and Methods

### 2.1. Sample Collection and Preparation

A hydrothermal chimney was collected from the Capelinhos site in the Lucky Strike hydrothermal field (37°17′21.90″ N, MAR) during the MoMARsat cruise in 2014 (doi: 10.17600/14000300) led by IFREMER (Plouzané France) onboard R/V *Pourquoi Pas?* [22]. The sample (PL583-8; http://video.ifremer.fr/video?id=9415, accessed on 29 October 2021) was collected and prepared in anaerobic conditions, as previously described, to inoculate the microbial electrochemical systems [23]. It is necessary to mention that the communities obtained in this study were enriched during the period 2016–2017.

### 2.2. Electrotrophic Enrichment in a Microbial Electrochemical System

An H-cell MES was filled with 1.5 L of sterile mineral medium, as previously described [24], with 0.1 g/L of Cysteine-HCl and supplemented with 4 mM nitrate, continuously flushed with N_2_:CO_2_ (90:10, 100 mL/min) to anaerobic conditions, and set at 80 °C and pH 6.0 throughout platform monitoring (see Supplementary data, Figure S1). The gases (H_2_, CO_2_, CH_4_, N_2_) evolution was followed as described in Pillot et al., 2018 [24]. The working electrode (cathode) composed of 20 cm^2^ of carbon cloth was poised at −590 mV vs. SHE using SP-240 potentiostat and EC-Lab software (BioLogic, Seyssinet-Pariset, France). The cathode served as the sole electron donor for the electrotrophs’ development. The system was inoculated with 8 g of the crushed chimney in anaerobic conditions. Current consumption was monitored via the chronoamperometry method, with current density and counter-electrode potential measurements taken every 10 s.

**Figure 1:**
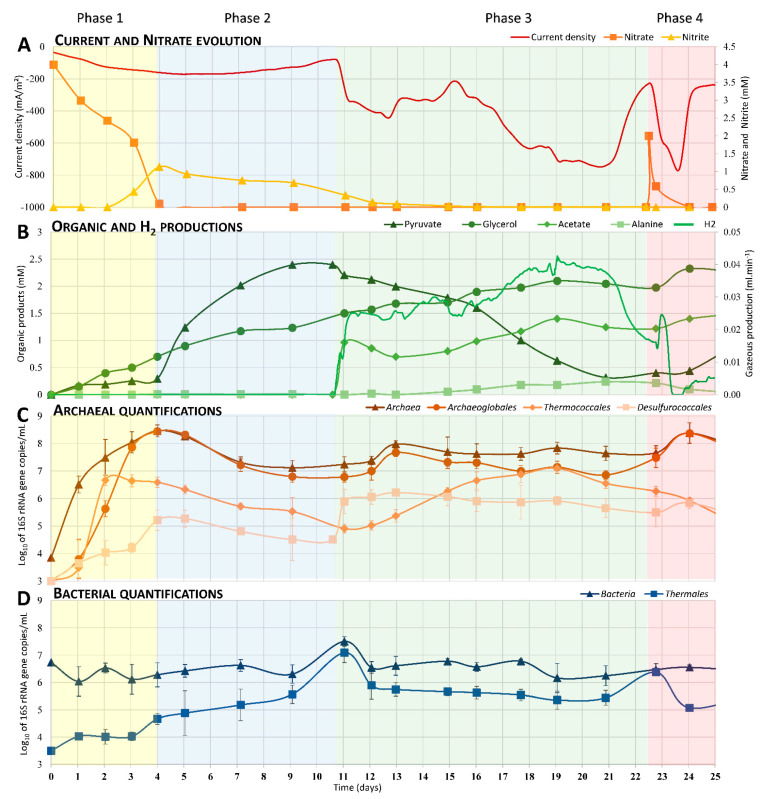
Consumption of nitrate (**A**), production of organic and H_2_ (**B**), and microbial 16S gene quantification (**C**,**D**) of nitrate-reducing eletrotrophic community enrichment culture (C1-experiment) over 25 days. A microbial electrochemical system at 80 °C was inoculated with ~0.5% (*w*/*v*) of a crushed chimney sample then incubated for 25 days. Each point corresponds to a sampling of the liquid medium during the growth of a biofilm on a polarized cathode at −590 mV vs. SHE. These samples were analyzed by qPCR, NMR, and HPLC. (**A**): current (mA∙m^−2^), nitrate, and nitrite (mM) evolutions. Nitrate (orange square) and nitrite (triangle yellow) were titrated by wet oxidation technique; (**B**): metabolic productions (mM) over time measured by HPLC and NMR. Pyruvate (green triangle), glycerol (green circle), acetate (green crystal), alanine (green square), H_2_ (green line); (**C**): qPCR evolution from Archaea specific primers. *Archaea* (brown triangle), *Archaeoglobales* (dark orange circle), *Thermococcales* (orange crystal) and *Desulfurococcales* (light orange square); (**D**): qPCR evolution from *Bacteria* specific primers. *Bacteria* (dark blue triangle) and *Thermales* (blue square).

In order to evaluate the enrichment of biodiversity over time on the cathode (biofilm) and in the liquid medium (LM, planktonic cells), three successive cultures were performed. For the first culture, shown in Figure 1, a fraction of a crushed chimney from the Capelinhos site was used to inoculate the MES. After 25 days of incubation, the cathode (C1-Biofilm) and the liquid medium (C1-LM) were harvested. An open circuit potential (OCP) control was performed in the same conditions. For the second culture, a new MES with a sterile electrode was inoculated with 150 mL of the C1-LM in a fresh mineral medium. The cathode (C2-Biofilm) and liquid media (C2-LM) were harvested after 7 days of enrichment when the current consumption stabilized. The third culture was performed with inoculation from 150 mL of C2-LM and enriched for 7 additional days. Abiotic control without inoculation did not show current consumption or nitrate depletion during the experimental period.

### 2.3. Nitrate/Nitrite Quantification

Nitrate consumption was analyzed using a wet oxidation technique with automated colorimetry, as described in [25], after culture medium centrifugation at 14,000 rpm for 5 min. The detection limit of this method is ~0.5 µM.

### 2.4. Identification and Quantification of Organic Compounds Production

To identify and quantify organics produced by the biofilm, samples of liquid media (10 mL) were collected every 24 to 48 h and analyzed by ^1^H NMR spectroscopy. Four hundred microliters of each culture medium was added to 200 µL of PBS solution prepared in D_2_O (NaCl, 140 mM; KCl, 2.7 mM; KH_2_PO4, 1.5 mM; Na_2_HPO4, 8.1 mM, pH 7.4) supplemented with 0.5 mM of trimethylsilylpropionic acid-d4 (TSP) as an NMR reference. All the 1D ^1^H NMR experiments were carried out at 300 K on a Bruker Avance spectrometer operating at 600 MHz for the ^1^H frequency and equipped with a 5 mm BBFO probe.

Spectra were recorded using the 1D nuclear Overhauser effect spectroscopy pulse sequence (Trd-90°-t1-90°-tm-90°-Taq) with a relaxation delay (Trd) of 12.5 s, a mixing time (tm) of 100 ms, and a t1 of 4 μs. The sequence enables an optimal suppression of the water signal that dominates the spectrum. One hundred twenty-eight free induction decays (FID) of 65,536 data points were collected using a spectral width of 12 kHz and an acquisition time of 2.72 s. For all the spectra, the FIDs were multiplied by an exponential weighting function corresponding to a line broadening of 0.3 Hz and zero-filled before Fourier transformation. NMR spectra were manually phased using the Topspin 3.5 software (Bruker Biospin Corporation, Billerica, USA) and automatically baseline-corrected and referenced to the TSP signal (δ = −0.015 ppm) using the Chenomx NMR Suite (version 7.5) software (Chenomx Inc., Edmonton, AB, Canada). A 0.3 Hz line-broadening apodization was applied prior to spectral analysis and ^1^H-^1^H TOCSY [26] and ^1^H-^13^C HSQC [27] experiments were recorded on selected samples in order to identify the detected metabolites. Quantification of identified metabolites was done using the Chenomx NMR Suite (version 7.5) software (Chenomx Inc.) using the TSP signal as an internal standard.

### 2.5. Biodiversity Analysis

The taxonomic affiliation was performed according to Zhang et al., 2016 [28]. DNA was extracted from 1 g of the crushed chimney and at the end of each period of culture from the scrapings of half of the working electrode (cathode) and from centrifuged pellets of 50 mL of spent media. The DNA extraction was carried out using the MoBio PowerSoil DNA Isolation Kit (Carlsbad, CA, USA). The V4 region of the 16S rRNA gene was amplified using the universal primers 515F (5′-GTG CCA GCM GCC GCG GTA A-3′) and 806R (5′-GGA CTA CNN GGG TAT CTA AT-3′) [29] with Taq&Load MasterMix (Promega). PCR reactions, amplicons sequencing, and taxonomic affiliation were carried out as previously described [24]. To analyze alpha diversity, the OTU tables were rarefied to a sampling depth of 13,190 sequences per library, and three metrics were calculated: the richness component (number of OTUs), Pielou’s index and Shannon’s biodiversity index [30]. Rarefaction curves are presented in Supplementary data, Figure S3. The raw sequences are available on the Sequence Read Archive (accession number: PRJNA734279-4-8, 12).

### 2.6. Microscopy Observation with Fluorescent In Situ Hybridization (FISH)

Prior to performing microscopic observations, the working electrode (cathode) from the end of each experiment was fixed with 2% paraformaldehyde and kept at 4 °C. To highlight the presence and abundance of microbes from different domains and orders, fluorescently labelled (CY3, FITC) probes (Biomers.net GmbH, Ulm, Germany) were used to label nucleic acids (Syto9), *Bacteria* (EUB338-FITC), *Archaea* (ARCH917-CY3), *Euryarchaeota* (Eury806), *Crenarchaeota* (Cren537), *Thermococcales* (Tcoc164) and *Archaeoglobales* (Arglo32) [31,32]. Cathodes were incubated with fluorescent probes in an equilibrated humidity chamber at 48 °C for 2–6 h and then washed with washing buffer (saline–sodium citrate buffer, pH7) at 42 °C for 15 min. Samples were then dried in 80% ethanol and mounted on a glass slide with antifadent AF1 (Citifluor, Hatfield, USA) added with 4′-6′-diamidino-2-phenylindole (DAPI) (Sigma Aldrich, St. Louis, MI, USA) for counterstaining at a final concentration of 2 µg·mL^−1^. Sample observations were performed on a confocal LSM780 microscope (Zeiss, Jena, Germany) equipped with a ×10, EC PLAN-NEOFLUAR objective. Green and red fluorescence emissions were acquired by excitation at 488 and 561 nm, respectively, using two lasers. Image stacks (at 0.5–1 µm steps) were acquired with GaAsP photomultiplier tube detectors. Epifluorescence micrographs were processed using the Zen software (Zeiss, Germany).

### 2.7. Quantitative PCR of Phylogenetic Orders

Quantification of *Bacteria*, *Archaea*, and specific phylogenetic orders retrieved in MiSeq analysis were carried out using a qPCR method. SsoAdvanced™ Sybr Green Supermix was used on a CFX96 Real-Time System (C1000 Thermal Cycler, Bio-Rad Laboratories, Hercules, CA, USA) with the primers specified in Table 1 [33,34]. 16S rRNA primers were specifically and manually designed for quantification of most represented orders using the MEGA6 software [35] and 16S rRNA reference sequences from the Silva database (http://www.arb-silva.de, accessed on 29 October 2021). Newly designed primers were validated in silico on the TestPrime software (https://www.arb-silva.de, accessed on 29 October 2021) and on different cultures of species belonging to and not belonging to the targeted order (see Supplementary information). The PCR program was composed of a 5 min initial denaturation step at 98 °C followed by 50 cycles of a 10 s denaturation step at 98 °C, a hybridization step of 20 s at the temperature indicated in Table 1, and a 40 s elongation step at 72 °C, with melting curves generated at the end of each reaction to ensure product specificity. A standard curve from 10^2^ to 10^10^ 16S rRNA gene copies was obtained by diluting pGEM-T plasmids harboring 16S rRNA gene fragments specific to *Bacteria*, *Archaea*, or specific members of each order.

qPCR quantifications were validated by comparison with microscopic counting on cultures of targeted species filtered on a 0.2 µm filter and stained with DAPI. Results of qPCR were expressed as number of 16S rRNA gene copies per milliliter of liquid media. Due to the qPCR protocol, a minimum threshold of detection of 3 log_10_/_mL_ was observed.

### 2.8. Principal Component Analysis

A PCA was performed on Rstudio (version 3.3.3) using the packages FactoMineR (v1.39), factoextra (v1.0.5), and corrplot (v0.84), and using a Spearman correlation.

## 3. Results

### 3.1. Current and Nitrate Consumptions

A preliminary characterization was carried out to control the growth conditions of the electroautotrophic biofilm. First, the organic matter present in the inoculum was measured by NMR, showing less than 75 µM accumulated of potential electron donors. An open circuit potential (OCP) control was then performed in the same conditions as enrichments and inoculated with the hydrothermal chimney. In this control, qPCR, NMR, and microscopy measurements didn’t show any growth over time (data not shown). Besides, in the MES filled with the mineral medium, the cathode poised at −590 mV vs. SHE was the only potential energy source available for microbial growth [19]. Under this experimental condition, CO_2_, continuously sparged into the reactor, was the only available carbon source, and nitrate, present in the mineral medium, was the only electron acceptor available (See supplementary data, Figure S1). The evolution of nitrate, nitrite, and current consumption are reported in Figure 1A. The nitrate reduction can be divided into two phases. During the first four days, the nitrate was consumed (from 4 mM to 0.22 mM), correlating with low production of nitrite (up to 1 mM). After D4 (Day 4), a slow consumption of nitrite (from 1 mM to 0.33 mM) was observed until D12. During these first two phases, a slow increase in current consumption was measured (up to −180 mA∙m^−2^) while nitrate and nitrite were consumed. A drastic increase in current consumption was then observed, reaching −750 mA∙m^−2^ by D20. At D22, the addition of 2 mM of nitrate allowed for a spike back to maximum current consumption (−765 mA/m^2^) before it quickly dropped again.

### 3.2. Electrosynthesis of Organic Compounds

During the growth of the nitrate-reducing electrotrophic community, different organic compounds were released in the liquid medium (Figure 1B), while none were detected in liquid media of the controls. Analysis of liquid samples over time using the NMR method allowed us to identify and quantify the production of a significant amount of acetate, glycerol, pyruvate, and alanine. Glycerol was continuously and slowly produced over time, reaching 2.3 mM at D24. Pyruvate started to be heavily produced at D4, reaching a maximum of 2.4 mM at D9 followed by a decrease (to 0.25 mM by D21) due to microbial consumption over time. This pyruvate production occurred following the total depletion of nitrate in the liquid medium. Acetate started to be produced at D11 to reach a maximum (1.4 mM on D19) correlated with the pyruvate decrease, the current consumption, and H_2_ production. Moreover, low production of alanine was detected after D11, reaching a maximum of 0.12 mM on D21 and decreasing thereafter.

H_2_ production (Figure 1B) appears to have been strongly related to the variation in current consumption (Figure 1A). Production increased quickly at D11 up to 0.025 mL.min^−1^, remained stable for five days, and then slowly increased to reach a maximum of 0.04 mL∙min^−1^ at D19. This production decreased from D21 on, correlated with the depletion of the pyruvate and leading to the decrease of current consumption (−220 mA∙m^−2^; D22).

Other organic compounds were produced during this experiment. Methanol and ethanol peaked at 36 µM and 0.18 mM, respectively, at D9 and D1 (data not shown). Moreover, slow production of acetamide (maximum of 69 µM at D12), benzoate-like molecule (slow increase up to 0.13 mM at D21), 2-aminoisobutyric acid (maximum of 67 µM at D1), and formate (varied between 8 and 70 µM between D1 and D21) were also observed over the experiment period (data not shown).

These H_2_ and organic compound productions were not observed in abiotic and OCP controls with nitrate, meaning that this production was catalyzed by the activity of microorganisms on the electrode.

### 3.3. Enrichment over Time of Dominant Phylogenetic Orders

To study the evolution of the composition of the electrotrophic community over time (Figure 1C,D), a quantification by qPCR was performed with primers specific to each significant order (Table 1). The quantifications were performed on harvested liquid samples (10 mL) over time. Due to the cathode’s status as sole energy source, planktonic microorganisms found in the liquid medium necessarily arose from on-electrode biofilm release (no growth in control conditions). During the first four days, a quick increase of archaeal 16S rRNA gene copies (Figure 1C) was observed (from 3.8 to 8.4 log_10_) at the same time as the four-day increase of *Archaeoglobales* (from <3 to 8.4 log_10_) and two-day increase of *Thermococcales* (from <3 to 6.6 log_10_). These increases corresponded to nitrate reduction and nitrite accumulation (Figure 1A). Between D3 and D4, *Desulfurococcales’* 16-rRNA-gene copies increased faster than the acceleration of the nitrate reduction and nitrite production. No increase in bacterial 16S-rRNA-gene copies was observed during this period. From D3 to D11, *Archaeoglobales, Desulfurococcales,* and *Thermococcales* 16S-rRNA-gene copies slowly decreased 1–1.5 log_10_ at the same time as the pyruvate accumulated in the liquid medium. During this period, the *Thermales* 16S-rRNA-gene copies increased (from 3.5 to 7 log_10_) in correlation with the nitrite reduction (Figure 1A,D). From D11 to D22, *Thermococcales*, and *Desulfurococcales* species were enriched while other orders slowly decreased in number or maintained their populations in the liquid media. The enrichment of the latter was correlated to the decrease in pyruvate and production of acetate, hydrogen, and alanine (fermentation products; Figure 1B). It is worth noting that *Archaeoglobales* species increased also between D11 and D13, correlating with the weak degradation of acetate (from 1 mM to 0.6 mM). Finally, during the last days of the experiment, the addition of nitrate allowed the enrichment of *Archaeoglobales* species followed by heterotrophic *Thermales*.

### 3.4. Statistical Analysis of the Correlation between Variables

**Figure 2:**
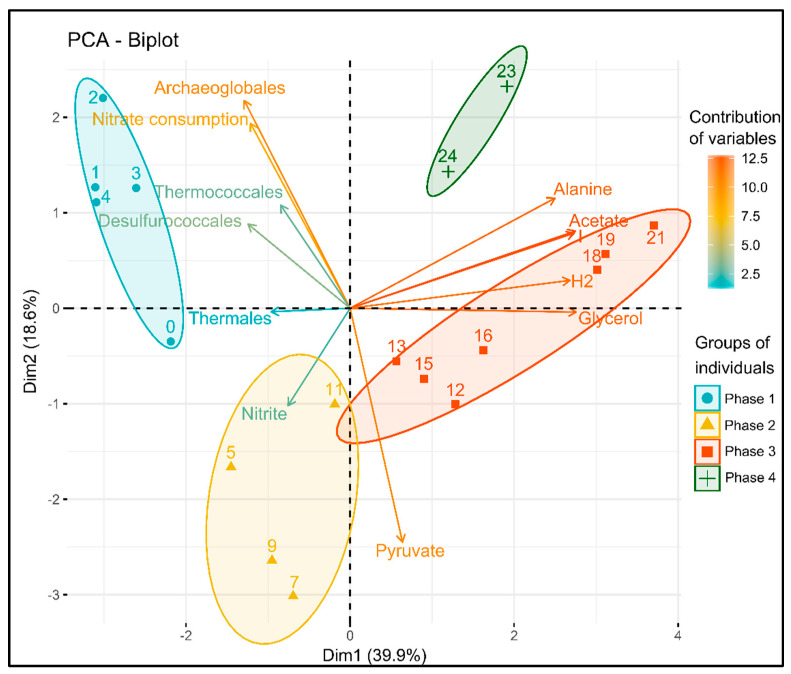
PCA of nitrate, nitrite, organic compounds, and evolution of quantity (qPCR) of dominant phylogenetic orders over the 25 days of C1-experiment culture. The biplot represents the distribution of each sample (characterized by the day number post-inoculation) and the contribution of each variable on the two first dimensions, explaining, respectively, 39.9% and 18.6% of the total variance. These two dimensions allowed for the discrimination of four temporal groups.

A principal component analysis (PCA) was performed to study the correlation between the current density; the nitrate, nitrite, and organic product concentrations; and microbial communities (Figure 2). It represents the distribution of each sample (represented by the day number postinoculation) and the contribution of each variable on a biplot composed of the two first dimensions, explaining, respectively, 39.9% and 18.6% of total variances.

These two dimensions allowed us to discriminate between four temporal groups, called phases, in this study. The first phase was represented by samples from D0 to D4 of culture, mainly explained by the *Archaeoglobales* evolution and the nitrate consumption but also inversely correlated to the pyruvate and fermentation products (Figure 2). The second phase (D5 to D11) was mainly characterized by the evolution of *Thermales,* nitrite, and accumulation of pyruvate. The third phase (from D12 to D22), mainly explained by the first dimension, was linked to the accumulation of fermentation products (alanine, acetate, H_2_) and current consumption. Finally, the fourth and last group, consisting of the samples from D23 and D24, was significantly different from the other groups but did not link specifically to some parameters. It is worth noting that the contribution of *Thermococcales* and *Desulfurococcales* was very weak in the building of the two dimensions of the PCA. Indeed, their evolutions could not be linked to one temporal group in the PCA. This can be explained by their evolution in more than one phase or independently of other variables.

### 3.5. Microscopic Observation of Electrotrophic Biofilm

**Figure 3:**
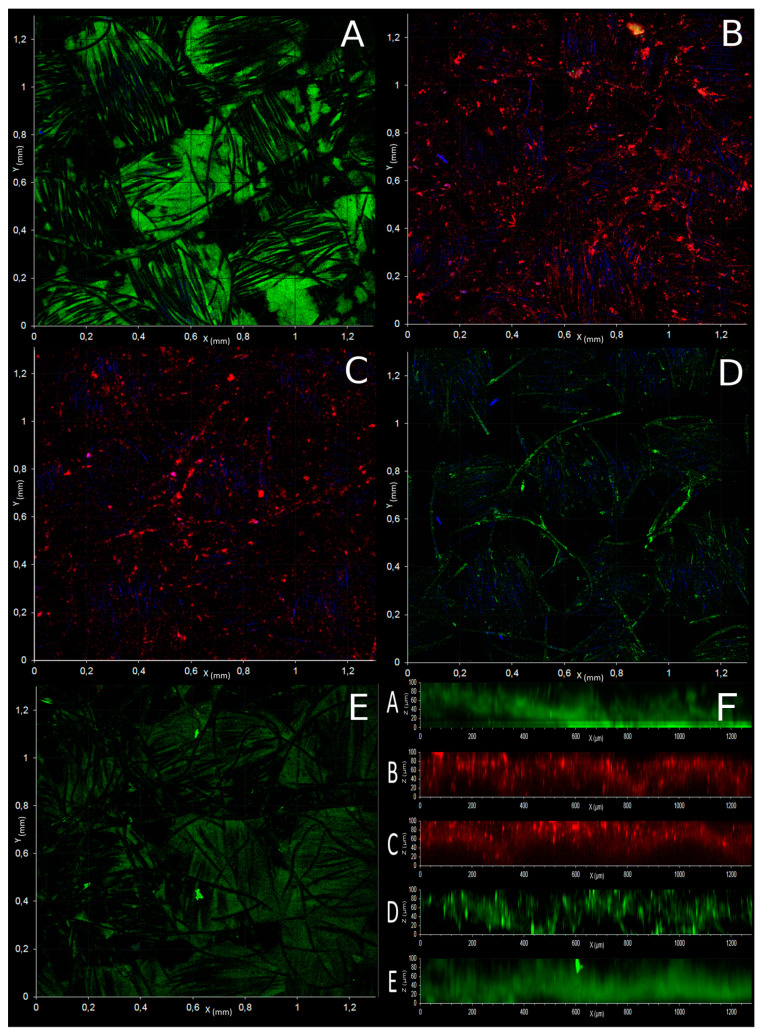
Confocal laser scanning microscope observations (**A**–**E**) and depth profile (**F**) of mature microbial biofilm developed on a cathode after 25 days incubation of C1-experiment (at ×100). Observations were realized on 3 cm^2^ cutting from the carbon cloth cathode. The biofilm on each piece of electrode was stained with one FISH probe (Table 1). (**A**): EUB338/FITC probe (green) specific to *Bacteria*, (**B**): Eury806/CY3 (red) specific to *Euryarchaeota*, (**C**): Cren537/CY3 (red) specific to *Crenarchaeota*, (**D**): Tcoc164/FTIC (green) specific to *Thermococcales*, and (**E**): Arglo32/FTIC (green) specific to *Archaeoglobales*. Blue signal corresponds to the reflectance of carbon fiber. Panel F shows the depth profile (orthogonal projection of x, z planes) of each FISH microscopy.

Confocal observation of electrotrophic biofilm on the cathode of the first experiment was performed with FISH probes specific to *Bacteria*, *Archaea*, *Euryarchaeota*, *Crenarchaeota*, *Thermococcales*, and *Archaeoglobales* (Figure 3). It allowed us to highlight the preferential presence of *Bacteria* (*Thermales*) and *Archaeoglobales* in the depth of the electrode (Figure 3A,E,F), while *Thermococcales* and *Crenarchaeota* (*Desulfurococcales*) (Figure 3C,D) were growing mostly on the external surface of the electrode. These observations suggest the colonization of nitrate-reducing microorganisms (e.g., some *Archaeoglobales* and *Thermales*) in the depth of the electrode while fermentative microbes (e.g., *Thermococcus* and *Thermodiscus* sp.) grew in the periphery of this colonization.

### 3.6. Biodiversity on Cathodes (Biofilms) and in Liquid Media (Planktonic Cells) of the Experiments

**Figure 4:**
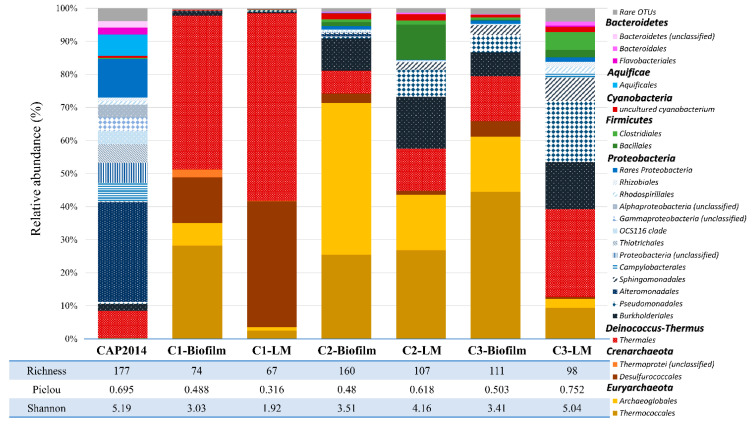
Dominant taxonomic affiliation at the order level (>1% of relative abundance) and biodiversity indices (Richness, Pielou and Shannon indices) of enriched microbial communities from a crushed chimney sample. These phylogenetic orders were enriched at 80 °C with a polarized cathode as the sole energy source (−590 mV vs. SHE) within a mineral liquid medium in presence of nitrate as electron acceptor. CAP2014: biodiversity initially present in crushed chimney sample, harvested from the Capelinhos site (Lucky Strike). C1-Biofilm: biodiversity of the first enriched biofilm after 25 days of incubation at 80 °C on a polarized cathode at −590 mV vs. SHE; C1-LM: biodiversity of the liquid medium of the first enrichment. C2-Biofilm: biodiversity of the enriched biofilm for 7 days in the same conditions after the subculturing of 150 mL from the liquid medium of C1 experiment; C2-LM: biodiversity of the liquid medium of the second subculture. C3-Biofilm: biodiversity of the enriched biofilm for 7 days in the same conditions after the subculturing of 150 mL from the liquid medium of C2 experiment; C3-LM: biodiversity of the liquid medium of the third enrichment. OTUs representing less than 1% of total sequences of the samples are pooled as “cRare OTUs”.

Once a first colonization of the cathode was obtained, we aimed to study if this community could lead to the development of a more complex and mature ecosystem over time, as observed in the older hydrothermal chimneys. Thus, two successive subcultures, C2 and C3 (cf. supplementary data, Figure S1), were performed in a new MES inoculated with media from the previous enrichment. After stabilization of current consumption, the communities of biofilms present on cathodes (C1-, C2- and C3-Biofilm) and in liquid media (C1-, C2- and C3-LM) were identified through 16S metabarcoding (Figure 4). While the environmental sample showed large biodiversity with a good relative evenness (Shannon index = 5.19 and Pielou’s evenness index = 0.695), the three enrichments showed an increase of the enrichment of microorganisms mainly in the liquid media (Shannon indexes: C1-LM = 1.92; C2-LM = 4.16, C3-LM = 5.04). The relative evenness in these enrichments showed an equally good distribution of species (evenness indexes: C1-LM = 0.316; C2-LM = 0.618; C3-LM = 0.752). However, the enrichment on the electrode remained equal across subcultures (Shannon index ~3.3 and evenness index ~0.49).

Based on average abundance analysis (Figure 4), the microbial diversity in the first culture (C1-Biofilm and C1-LM) was dominated by species belonging to four orders: *Thermococcales* (28.2%, 2.5%), *Archaeoglobales* (6.8%, 1%), *Desulfurococcales* (13.9%, 38.1%), and *Thermales* (46.5%, 56.8%), respectively, on the cathode and in the liquid medium. The remaining biodiversity represented only 0.46% on the electrode and 0.16% in the liquid media, shared between *Proteobacteria*, *Firmicutes*, and *Actinobacteria* species.

The second culture (C2-Biofilm and C2-LM) showed specific enrichment of *Archaeoglobales* on the electrode (45.8%) and in the liquid medium (16.8%). Moreover, the *Thermococcales* remained stable on the electrode (25.5%) while they were enriched in the liquid medium (26.8%). By contrast, the proportions of *Desulfurococcales* (2.8%, 1.2%) and *Thermales* (6.85%, 12.7%) drastically dropped on the electrode and in the liquid medium. Interestingly, we observed an increase of the remaining biodiversity with the enrichment of *Burkholderiales* (9.75–15.64%), *Pseudomonadales* (8.22% only in liquid), and *Bacillales* (10.7% only in liquid) in this second subculture.

Finally, the last enrichment (C3-Biofilm and C3-LM) showed the massive growth of *Bacteria* in the liquid media (87.3%), mainly affiliated to *Thermales* (26.4%), *Burkholderiales* (14.3%), *Pseudomonadales* (18.4%), *Sphingomonadales* (6.9%), and *Clostridiales* (5.46%), while the composition of the biofilm on the cathode was substantially the same as in the second enrichment. The taxonomic affiliation of dominant OTUs, by BLAST, has allowed us to identify the *Thermococcales* as closely related to around 20 *Thermococcus* spp., *Archaeoglobales* to *Geoglobus ahangari* strain 234 (99% similarity), *Archaeoglobus* sp. Fe70 (99% similarity), and *Ferroglobus placidus* (98% similarity), and *Desulfurococcales* to *Aeropyrum* sp. AF1T6.18 (98% similarity) and *Thermodiscus maritimus* (97% similarity). For *Bacteria*, *Thermales* were composed of 2 OTUs, mainly affiliated to *Vulcanithermus mediatlanticus* strain TR (99% similarity) and a new species of *Thermaceae* (close, at 95% similarity, to *Vulcanithermus* sp. BF2T511), respectively, *Bacillales* to *Geobacillus thermodenitrificans*, *Burkholderiales* to *Ralstonia* spp., and *Pseudomonadales* to *Pseudomonas* spp.

## 4. Discussion

The aim of this study was to follow the evolution of a microbial community and identify the first colonizers, growing on a cathode (at −590 mV vs. SHE), using nitrate as electron acceptor and in autotrophic conditions, after inoculation with a hydrothermal chimney sample. In these conditions, the presence or not of potentially low H2 production at the cathode was largely discussed [19]. In any case, the microorganisms growing from electrons provided by the polarized cathode (no growth in open circuit potential) were considered as electroautotrophs, whether through direct or partially H_2_-mediated electron transfer at the nanoscale (enhanced or not by the production and deposition of free cell-derived enzymes, on the cathode [36]).

**Figure 5:**
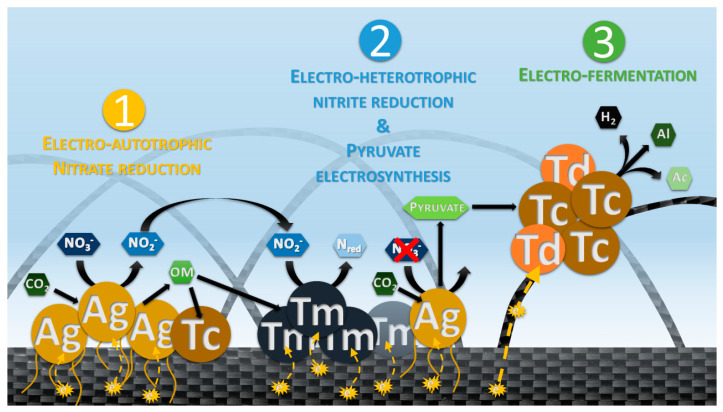
Schematic representation of the trophic chain model obtained from the monitoring of electrotrophic community enrichment in the presence of a cathode as an electron donor and nitrate as an electron acceptor. In this trophic chain, the *Archaeoglobales* are the first colonizers by reducing nitrate into nitrite and fixating CO_2_ into organic matter during the first phase. The latter is then used during the second phase by heterotrophs for growth, through fermentation for the *Thermococcales* and denitrification for the *Thermales*. When the nitrate is depleted, *Archaeoglobales* produce a high quantity of pyruvate through electrosynthesis. Afterward, *Thermococcales* and *Thermodicus* consume the pyruvate produced in combination with electrons from the cathode by electrofermentation, during the third phase. In the fourth phase, not represented here, the addition of nitrate allows the already developed communities to perform all these metabolisms at the same time. OM = Organic matter, Ag = *Archaeoglobales*, Tc = *Thermococcales*, Tm = *Thermales*, Td = *Thermodiscus*.

In these conditions, the increase of the current consumption, the decrease and transformation of the electron acceptor, the production of organic intermediates, as well as the deduction of the metabolisms involved (from closest relatives) and the evolution of the community are proof of the development of a hyperthermophilic electroautotrophic community on a cathode. To follow the community on an electrode, qPCR of the main phylogenetic groups was performed on the planktonic phase, as proxy, as the only possible source of planktonic cells was through the trophic chain initiated by electroautotrophic microorganisms at the cathode. To further identify the first colonizers of this community and the metabolisms involved, an analysis of the correlations between the different parameters (qPCR, organic compounds, electron acceptor) was performed. The PCA (Figure 2) and the observations of the evolution of the parameters (Figure 1) allowed us to separate the experiment into four phases, summarized in Figure 5:

### 4.1. Phase 1: Electrotrophic Hyperthermophilic Nitrate Reduction

The first phase, from inoculation to D4 (Figure 1A), corresponds to the nitrate reduction with a transient nitrite accumulation while current is consumed. This is related to the growth of *Archaeal* species, mainly *Archaeoglobales* immediately followed by *Thermococcales* and then *Desulfurococcales* species (Figure 1C). Alongside this growth, low production of organics is observed, mainly glycerol and pyruvate. Some species of the order *Archaeoglobales* (*Geoglobus ahangari* and *Ferroglobus placidus*) have already been described to perform extracellular electron transfers by using an electrode as electron acceptor [37]. How Archaea carry out exogenous electron transfer is still unknown [38], but the electrotrophic metabolism is recognized when an increase of current consumption is observed [36,39]. As no soluble electron donors or organic compounds are present at the initial time after inoculation (measured by NMR, HPLC, and µGC), only electroautotrophs can grow. Among the three enriched orders, *Archaeoglobales* are the only species known to grow from CO_2_ as carbon source and an inorganic electron source (provided by the cathode in this study). The three genera, belonging to *Archaeoglobales* orders, are known to grow chemolithoautotrophically through the reductive acetyl-CoA/Wood-Ljungdahl pathway, and their abilities to reduce the nitrate has already been observed (*Ferrogolbus* spp.) or is presumed (*Geoglobus*, *Archaeoglobus* spp.) [40,41,42,43]. Tests on pure cultures of *Geoglobus ahanghari* on cathodes polarized at −600 mV vs. SHE have confirmed its ability to reduce nitrate (unpublished data). The growth of the autotrophic *Archaeoglobales* species allowed for the production of biomass and the release of organic compounds from CO_2_ reduction during the first four days. These compounds then served as a source of carbon and energy for the various heterotrophic microorganisms that subsequently developed.

In addition, *Thermococcales* increased during the first three days (Figure 1C). All members of *Thermococcales* are characterized by their ability to ferment complex or simple peptides as an energy and carbon source by using elemental sulfur as an electron acceptor [44]. The breakdown of peptides and amino acids leads to the subsequent production of organic acids linked to substrate-level phosphorylation. Many *Thermococcales* species can also grow by fermentation of various carbohydrates without the need for S° [45]. The major fermentation products are acetate, H_2_, and CO_2_. Finally, some *Thermococcales* species have been described as carboxydotrophic, using the oxidation of carbon monoxide (CO) as an energy source and a source of organic carbon in the form of a peptide [46,47,48,49], with H_2_ and CO_2_ as major products of the metabolism.

These various fermentative metabolisms allowed for the growth of *Thermococcales* species from organic carbon sources supplied by the growth of *Archaeoglobales* during the first three days.

Alongside *Archaeoglobales* and *Thermococcales* growth and nitrate consumption, the growth of heterotrophic *Desulfurococcales* and *Thermales* has been observed (Figure 1C,D). While only one species of *Desulfurococcales*, *Pyrolobus fumarii,* has been shown to reduce nitrate [50], most of *Thermales*, such as *Vulcanithermus* spp. or *Oceanithermus* spp., are able to reduce nitrate and in some cases even nitrite [51]. The delay before *Desulfurococcales* and *Thermales* emergence suggests their heterotrophic growth from syntrophic relations. These trophic relations would have been set by (i) fermentation of organics produced, (ii) direct interspecies electron transfer (DIET), and/or by (iii) partially performing the last step of the dissimilatory nitrate reduction from nitrite produced by nitrate-reducing microorganisms.

### 4.2. Phase 2: Electrosynthesis of Pyruvate

The second phase is marked from D4 by the increase of pyruvate production (Figure 1B), the complete depletion of nitrate, the accumulation of nitrite, and the stopped growth of *Archaeoglobales*. However, *Archaeoglobales* species self-sustained for a period of several days (D4 to D11) with a new type of energetic coupling that remains to be fully explored. The inflexion of pyruvate accumulation is correlated to the lack of an electron acceptor. As previously mentioned, the *Archaeoglobales* species use the reductive acetyl-CoA pathway for carbon fixation through the bifunctional CODH/acetyl-CoA synthase complex [52]. Pyruvate and Acetyl-CoA are the main intermediates that can be used directly for biosynthesis [53]. This assimilation of CO_2_ into pyruvate and acetyl-CoA requires reduced electronic shuttles (NADH, NADPH, Fdred; [54]). The pyruvate is then normally used for biosynthesis through conversion to oxaloacetate by pyruvate carboxylase [55], which uses one mole of ATP regenerated through the respiratory/energetic metabolism (by respiring on nitrate in this study for example).

However, in the absence of an electron and subsequent ATP regeneration, the excess of CO_2_ as well as electrons continuously injected into the cells through the cathode (−590 mV vs. SHE) would lead to the accumulation of pyruvate. For *Thermococcales*, their growth stopped in the D2 to D11 period, though they are known to be able to perform pyruvate fermentation to acetate through the oxidative decarboxylation of the pyruvate by the pyruvate:ferredoxin oxidoreductase (POR) [55,56]. However, nitrite is known to cause the inhibition of POR in *Thermococcales* [56], which would explain why pyruvate is not consumed throughout this period (D4 to D11).

### 4.3. Phase 3: Electrofermentation of Pyruvate

The third phase of our experiment (D11 to D22.5) was characterized by an increase in current consumption, the complete depletion of nitrite, and the fermentation of pyruvate into acetate, alanine, and H_2_ (Figure 1A,B). During these phenomena, *Thermodiscus* and *Thermococcus* populations increase in liquid media. *Thermodiscus* species are poorly studied but are described as obligatory heterotrophic *Crenarchaeota* capable of sulfur respiration and fermentation of complex organic compounds [57]. *Thermococcales* are also known to be heterotrophic and fermentative *Euryarchaeota*. Thus, *Thermococcus* and *Thermodiscus* started to grow through pyruvate fermentation only after nitrite was depleted on D11, producing acetate, alanine, and H_2_ [58]. This fermentation from D11 was related to current consumption, suggesting an electrofermentative process. Little is known about the mechanisms involved in electrofermentation and the distribution of this ability in the prokaryotic phylogenetic groups. Further investigation on this putative mechanism in *Thermococcus* and *Thermodiscus* is necessary to surely link this current consumption and fermentation. Interestingly, the FISH microscopic observations (Figure 3) have shown the peripheral development of *Thermococcales* and *Desulfurococcales* on the electrode around *Archaeoglobales*, suggesting a successive development over time. This spatial configuration allowed direct contacts between the electrode, *Archaeoglobales,* and fermentative microorganisms. Thus, this increase of current consumption could be attributed to a mechanism of direct interspecies electron transfers (DIET) already observed in various cocultures [59,60]. In this hypothesis, *Thermococcales* served as an electron acceptor for *Archaeoglobales*, allowing them to grow again in the absence of nitrate while also supplying *Thermococcales* with electrons for their metabolism. The electron flow introduced by *Archaeoglobales* to *Thermococcales* would permit or contribute to the electrofermentation of pyruvate, allowing for the concomitant growth of *Archaeoglobales* and *Thermococcales* through electron transfer (D11 to D13). But this potential explanation remains hypothetical and requires further test in cocultures to corroborate it.

### 4.4. Phase 4: The Trophic Chain from Electroautotrophic Nitrate Reduction to Heterotrophy

In the fourth and last phase, the successive addition of nitrate after D22 (Figure 1) showed the simultaneous activation of the different metabolisms with the consumption of nitrate, increase of heterotrophic and then autotrophic nitrate-reducing microorganisms, alongside the production of fermentation products (H_2_ and acetate). This indicates that in mature communities, metabolic plasticity is developed to allow trophic interaction between auto- and heterotrophs but also respiring (including potential DIET) and fermentative microorganisms to allow their mutual survival in harsh and quickly changing conditions in a hydrothermal context.

### 4.5. Thereafter: Development of More Complex Ecosystems over Subcultures

After the fourth phase, two successive subcultures on cathodes were performed with the harvested liquid media of the previous enrichment. Inoculums only brought less than 0.3 mM of accumulated organic products (acetate, glycerol, and pyruvate) and 7 µg of estimated biomass (qPCR × mass of a cell). The MiSeq results exhibited the enrichment of a part of the biodiversity initially present in the inoculum (Figure 4). Subcultures of electrotrophic biofilm from successive planktonic cells allow for the development over time of more and more diverse species in liquid media and on the cathode. These late-growing species are affiliated to *Proteobacteria*, mainly uncultured members of *Burkholderiales*, *Pseudomonadales,* and *Sphingomonadales*. Members of these groups were previously isolated from hydrothermal vents [61,62,63]. These bacterial orders are only represented by heterotrophic species. Thus, their late development after the autotrophic condition of the experiment showed trophic interaction between electroautotrophic and heterotrophic species.

## 5. Conclusions

As supposed by Yamamoto et al., 2017 [10], electricity generation in deep hydrothermal systems is expected to affect surrounding biogeochemical processes and the development of microbial communities. Here, we have demonstrated that some microorganisms are able to use the energy of a polarized electrode as an alternative energy source to other and conventional electron donor molecules. Thus, this source of electronic energy would be one of the easily accessible energy means for these facultative electroautotrophic microorganisms, particularly those belonging to the *Archaea* domain. In addition, the probable presence of DIET within the studied microbial populations allows for the extrapolation of these results on a metabolic alternation in these ecosystems, which would lead these electroactive species to maintain or grow according to the presence or absence of an electron acceptor. This is especially true since the constant electron flow provided by this electrical current is more stable and reactive than the fluctuant emissions of H_2_ or CH_4_ from the hydrothermal fluid. In perspective, we can ask the question of the importance of this electric current on the origin of life. Could this electrical source have served as an energetic driving force allowing the slow prebiotic synthesis reaction in a delimited space (organic micelles or mineral microcavity) to form the protometabolism and the protocell?

## Figures and Tables

**Table 1:** 16S rRNA primer for qPCR quantification of *Bacteria*, *Archaea*, specific phylogenetic orders, and 16S rRNA labelled probes for FISH microscopy. The validation of the qPCR primers developed in this study are presented in the supplementary information: validation qPCR probes.

Phylogenetic Groups				
Target	Name	Sequence	T_hyb_	Ref.
*Bacteria*	GML5F	GCCTACGGGAGGCAGCAG	55 °C	[34]
	Univ516	GTDTTACCGCGGCKGCTGRCA		
*Archaea*	Arc931F	AGGAATTGGCGGGGGAGCA	62 °C	[33]
	m1100R	BTGGGTCTCGCTCGTTRC C		
*Thermococcales*	Tcoc_F959	CGTGAGGCGTCCACTTAAGTGTGGT	63 °C	This study
	Tcoc_R1233	GATGATGACRCGCGGGTACTAGGG		
*Archaeoglobales*	Arglo_F1077	CGGGCAACGGCAGGTCCGTATG	62 °C	
	Arglo_R1191	GTTGCAGCCCTCGATCCCAGGT		
*Desulfurococcales*	Univ516F	TGYCAGCMGCCGCGGTAAHAC	61 °C	
	DSC_748R	AACASYTAGCCCGCATCGTTTACAGCC		
*Thermales*	Therma/Deino_341F	GGAGGCAGCAGTTAGGAATCTTC	59 °C	
	Univ516R	GTDTTACCGCGGCKGCTGRCA		
**Fish Probes**				
**Target**	**Name**	**Sequence**	**Label**	**Ref.**
*Bacteria*	EUB338	GCT GCC TCC CGT AGG AGT	FITC	[31,32]
*Archaea*	ARCH917	GTG CTC CCC CGC CAA TTC	CY3	
*Euryarchaeota*	Eury806	CAC AGC GTT TAC ACC TAG	CY3	
*Crenarchaeota*	Cren537	TGA CCA CTT GAG GTG CTG	CY3	
*Thermococcales*	Tcoc164	CAV RCC TAT GGG GGA TTA GC	FITC	
*Archaeoglobales*	Arglo32	TTA GTC CCA GCC GGA TAG CA	FITC	

## Data Availability

All sequence data were deposited in the National Center for Biotechnology Information (NCBI) Sequence Read Archive (SRA) database under accession number PRJNA734279: 4-8, 12.

## Supplementary Materials

The following are available online at https://www.mdpi.com/article/10.3390/microorganisms9122475/s1, Figure S1: Summary of the experimental protocol of the study, Figure S2: Putative electrosynthesis pathway through the Wood-Ljungdahl carbon fixation pathway, Figure S3: Rarefaction curves of the 16S rRNA gene sequencing with universal primers 515F-806R. Supplementary information: Validation qPCR probes.
