# Supplementary material for: Evolution of Thermophilic Microbial Communities from a Deep-Sea Hydrothermal Chimney under Electrolithoautotrophic Conditions with Nitrate
Source: Microorganisms. 2021 Nov 30;9(12):2475. doi: 10.3390/microorganisms9122475 (PMC8705573; doi:10.3390/microorganisms9122475)
Supplement: Supplementary file 1 [file microorganisms-09-02475-s001.zip › Supplementary Files/Figures S1-S3_Supplementary data.pdf]

Evolution of thermophilic microbial communities from a deep-sea hydrothermal chimney under electrolithoautotrophic conditions with nitrate.

Guillaume Pillot[1], Oulfat Amin Ali[1], Sylvain Davidson[1], Laetitia Shintu[2], Yannick Combet-Blanc[1], Anne Godfroy[3], Patricia Bonin[1], Pierre-Pol Liebgott[1*]

[1] Aix Marseille Université, IRD, Université de Toulon, CNRS, MIO UM 110, Marseille, France

[2] Aix Marseille Université, Centrale Marseille CNRS, iSm2 UMR 7313, 13397, Marseille, France

[3] Laboratoire de Microbiologie des Environnements Extrêmes, Université de Bretagne Occidentale, CNRS, IFREMER, 29280 Plouzané, France.

* Pierre-Pol Liebgott. **Email:** pierre-pol.liebgott@mio.osupytheas.fr

Keywords: Electrosynthesis, Electrotrophy, Thermophily, Deep-sea hydrothermal vent, Nitrate

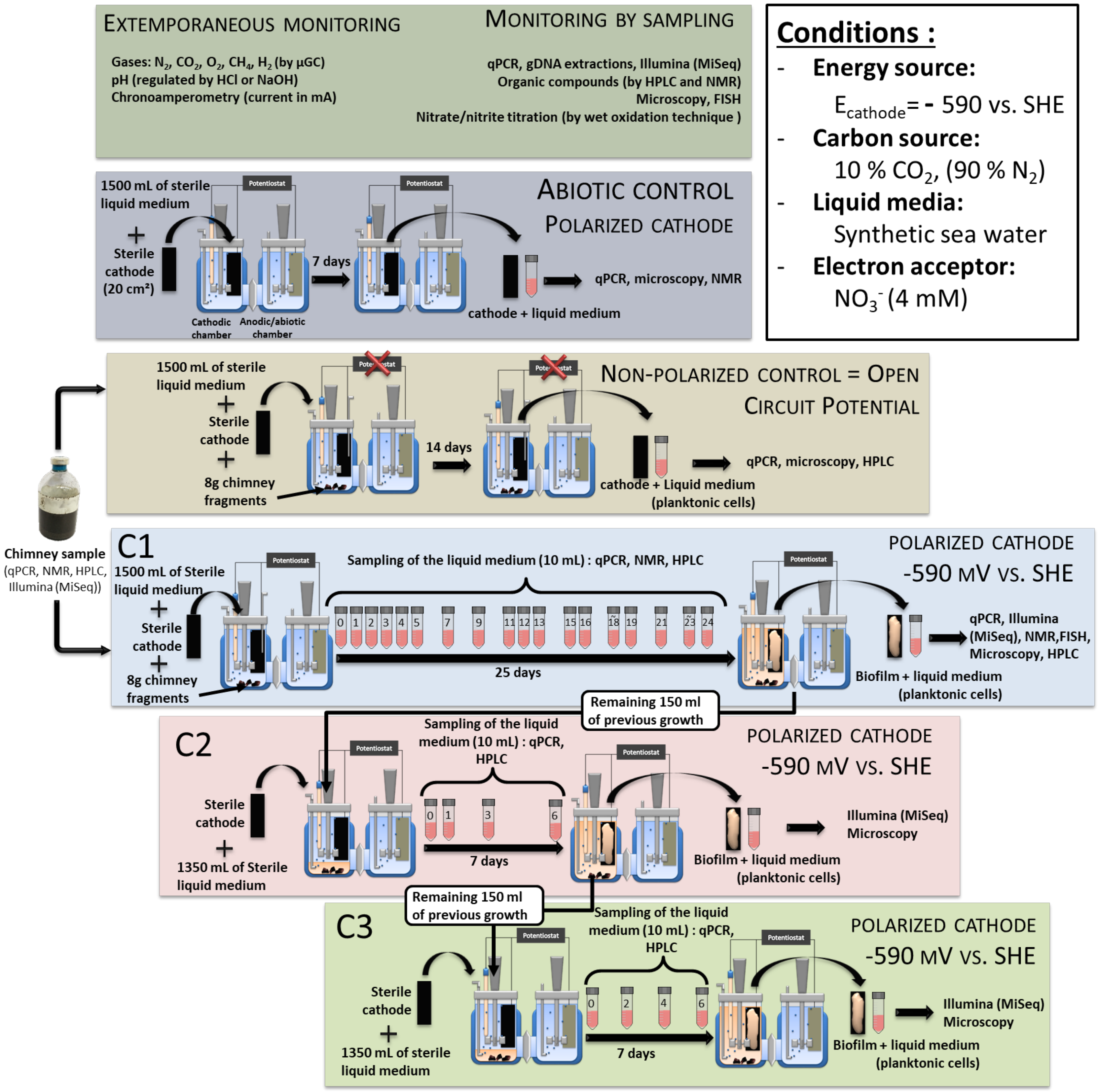

Fig. S1: Summary of the experimental protocol of the study

Liquid medium composition: 30 g/L NaCl, 0.65 g/L KCl, 0.5 g/L NH4Cl, 0.3 g/L KH2PO4, 0.3 g/L K2HPO4, 0.1 g/L MgCl2, 0.1 g/L CaCl2, 0.1 g/L Cys-HCl, and 10 mL/L of a Balch solution of trace elements. The incubation temperature was 80°C and the pH was regulated at 6.0. Two types of control experiments were performed in the laboratory. The first abiotic control contained a polarized cathode immersed in a sterile liquid medium without any biological inoculum. After incubation of 7 days, the electrode (cathode) and liquid medium were harvested and analyzed by qPCR, microscopy and HPLC. They showed no growth or any current consumption. Moreover, no compounds produced were observed other than cystine (resulting from the oxidation of cysteine). The second control experiment was an open circuit potential condition, i.e., with an unpolarized electrode. The liquid medium was inoculated with 8 g of the crushed chimney and growth was monitored for 2 weeks. No products (HPLC analyses) and no growth (microscopic observations) were observed during these 2 weeks of incubation. Next, experiment C1 was performed in a sterile liquid medium inoculated with 8 g of crushed chimney and with the cathode polarized at -590 mV vs. SHE. During 25 days of incubation, 18 samples were collected (10 mL) and then analyzed by qPCR, HPLC and NMR allowing an extemporaneous follow-up of specific microbial communities. After 25 days of incubation, the electrode (C1-Biofilm) and the liquid medium (C1-LM) were harvested. For the second culture (C2), a new MES with a sterile electrode was inoculated with 150 mL from C1-LM in a fresh mineral medium. The electrode (C2-Biofilm) and liquid medium (C2-LM) were harvested after 7 days of enrichment when the current consumption stabilized. The third culture (C3) was performed with the inoculation of 150 ml of C2-LM and enriched for an additional 7 days.

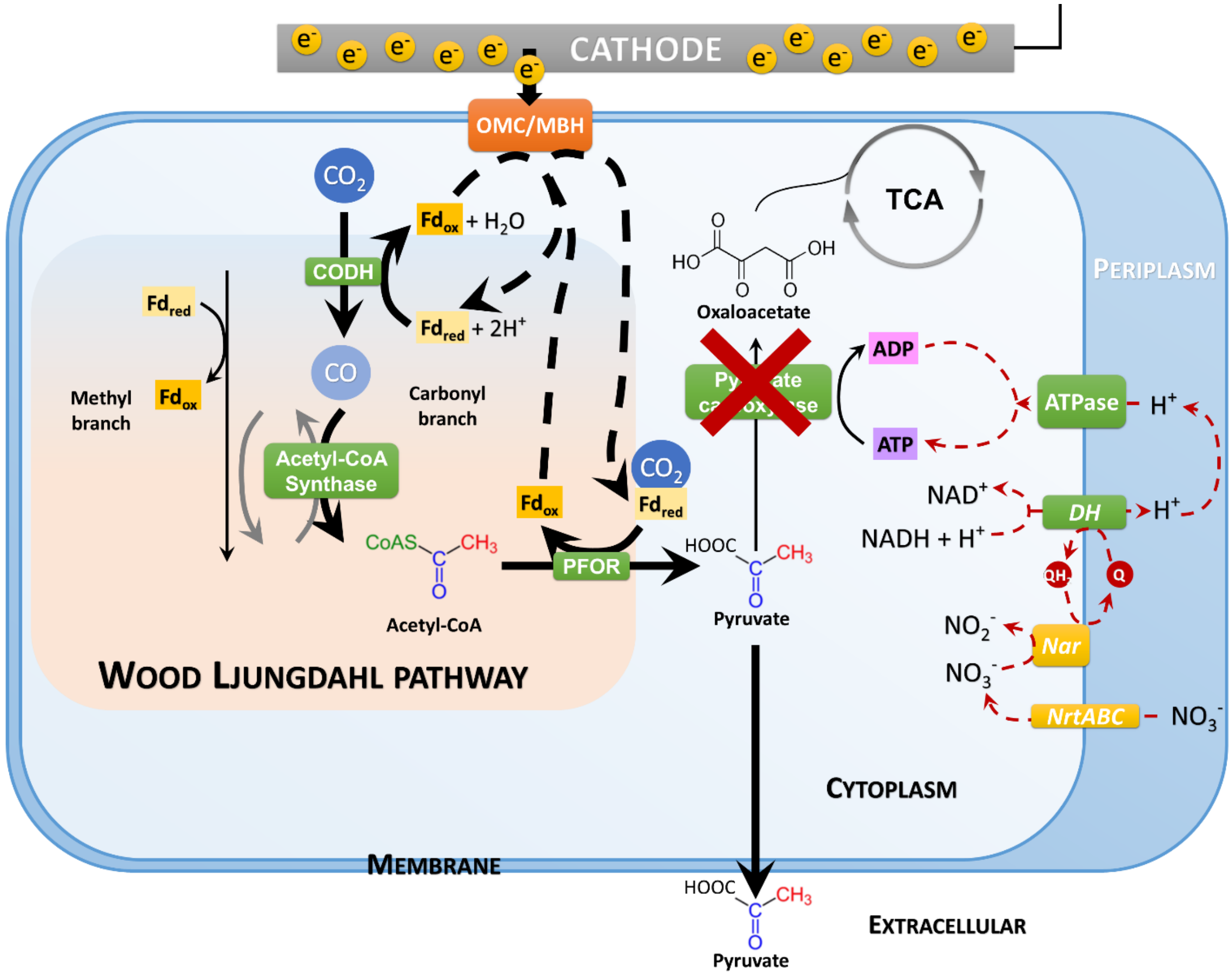

Fig. S2. Putative electrosynthesis pathway through the Wood-Ljungdahl carbon fixation pathway.

Red dot lines represent the hypothesis of the inhibition of those reactions in the absence of electron acceptor and an overfeeding of electrons (conductive chimney walls or polarized cathode). Fd_{red}: reduced ferredoxin; Fd_{ox}: oxidized ferredoxin; CODH: Carbon monoxide dehydrogenase; PFOR: Pyruvate dehydrogenase; NarABC: nitrate permease, Nar: nitrate reductase; DH: NADH dehydrogenase complex; TCA: tricarboxylic acid cycle. This putative pathway highlights that in excess of electron donor, here electrons from the cathode, the cytochromes or hydrogenase involved in the electron transfer led to an over-regeneration of reducing equivalents such as Ferredoxin. These reducing equivalent are involved in the Wood Ljungdahl pathway converting CO_2 up to Pyruvate. The Pyruvate is then incorporated in the TCA cycle by its conversion into Oxaloacetate by the Pyruvate carboxylase. This step requires ATP. In absence of Nitrate, the enzymes of Nitrate reduction can't produce the Quinone pool leading to the delocalization of protons and the regeneration of ATP by the ATPase. Then, an accumulation of Pyruvate is expected in this condition.

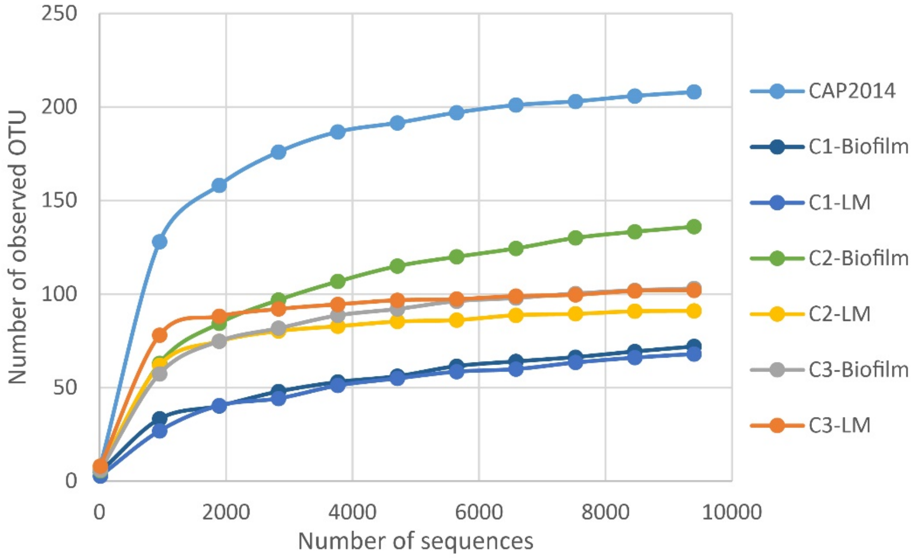

Fig. S3: Rarefaction curves of the 16S RNA gene sequencing with universal primers 515F-806R.
